# Successful removal of giant mediastinal lipoma and liposarcoma involving both chest cavities: Two case reports

**DOI:** 10.1097/MD.0000000000011806

**Published:** 2018-08-10

**Authors:** Chen Chen, Mingjiu Chen, Wenliang Liu, Yunchang Yuan, Fenglei Yu

**Affiliations:** Department of Thoracic Surgery, The Second Xiangya Hospital, Central South University, Changsha, Hunan, China.

**Keywords:** lipoma, liposarcoma, mediastinal tumor, sternotomy, surgery

## Abstract

**Rationale:**

Surgical removal of a giant mediastinal lipoma or liposarcoma involving both chest cavities is always challenging.

**Patient concerns:**

We present 2 cases of giant mediastinal tumor, one of which was a 22-year-old female who was admitted to our hospital due to a mild dyspnea after running. Computed tomography (CT) scan revealed a large mass with low density occupying the entire right hemithorax and extending anteriorly into the left. The other patient was a 43-year-old male, who was presented to the hospital with complaints of gradually progressive dyspnea. CT scan revealed a mass comprised of fat density with areas of soft-tissue density in-between, involving in both chest cavities, draping around the heart and great vessels.

**Interventions:**

Both of the patients receive complete resection through a standard median sternotomy.

**Diagnoses:**

Histologic examination revealed lipoma for the first patient, and well differentiated liposarcoma for the second.

**Outcomes:**

Both of their symptoms were improved after surgery and the postoperative courses were good.

**Lessons:**

Our experience indicated that complete surgical removal through a standard median sternotomy is a safe and efficient approach for the treatment of giant mediastinal lipoma and liposarcoma.

## 1 Introduction

Lipomas are very common benign neoplastic mesenchymal tumors arising from adipose tissue, while liposarcomas are the most common soft-tissue sarcomas in adult.^[1–4]^ However, giant mediastinal lipoma/liposarcoma involving both hemithorax, resulting in the compression of the lung with attendant respiratory embarrassment, is extremely rare in clinic.^[2,3,5]^ With good outcomes, surgical removal remains the first choice for the treatment of these kinds of diseases. Nevertheless, the management of a very huge mediastinal tumor involving both chest cavities is always challenging.^[4–6]^ Herein, we present our experiences on the surgical management of 2 cases of giant mediastinal tumors, one of which was lipoma and the other one was liposarcoma.

## 2 Case reports

**Figure 1:**
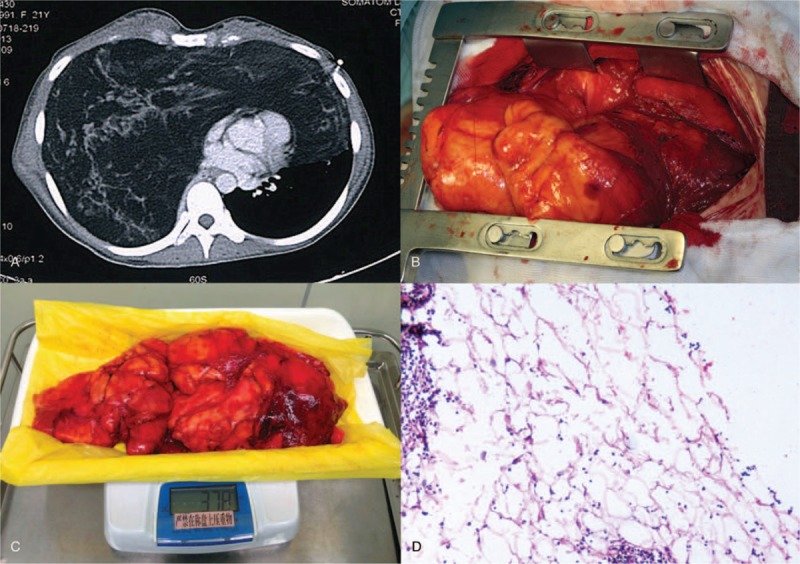
(A) Computed tomography scan revealing a large mass with low density occupying the entire right hemithorax and extending anteriorly into the left hemithorax. (B, C) The tumors were approximately 40 × 25 × 15 cm in size, 3.78 kg in weight. (D) The histologic examination of the surgical specimen confirmed the diagnosis of lipoma.

The 1st patient was a 22-year-old female, who was admitted to our hospital with complaint of a mild dyspnea after running. The patient had no history of cough, loss of weight, hemoptysis, or other constitutional symptoms. Physical examination showed the right lung field was dull to percussion with decreased breath sounds on auscultation. Chest computed tomographic (CT) scan revealed a large mass with low density occupying the entire right hemithorax and extending anteriorly into the left hemithorax, causing mediastinal shift and lung collapse (Fig. 1A).

**Figure 2:**
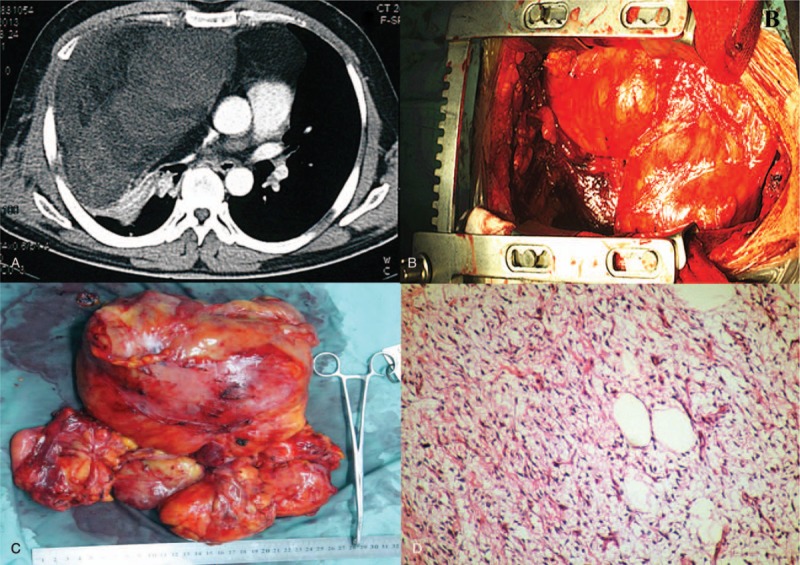
(A) Computed tomography scan revealing a mass comprised of fat density with areas of soft-tissue density in-between, involving in both chest cavities. (B, C) The tumors were approximately 28 × 25 × 10 cm in size, 2.85 kg in weight. (D) The histologic examination of the surgical specimen confirmed the diagnosis of liposarcoma.

The other patient was a 43-year-old male, who was presented to the hospital with complaints of gradually progressive dyspnea for 4 months. The patient had no history of cough, hemoptysis, loss of weight, or other constitutional symptoms. Physical examination was similar to the first patient. CT scan revealed a mass comprised of fat density with areas of soft-tissue density in-between, involving in both chest cavities, draping around the heart and great vessels (Fig. 2A).

For both patients, bronchoscopy, head magnetic resonance imaging, abdominal ultrasound, and a bone nuclear scan confirmed no involvement by the tumor. Blood biochemistry and serum cancer markers were all normal.

To remove the giant mediastinal mass, both of the patients were explored through a sternotomy to allow complete access to both pleural cavities and the mediastinum. The tumors were visualized crossing the anterior mediastinum and extending into both thoracic cavities (Figs. 1B and 2B). The gross appearance of the tumors were pale yellow, soft, with smooth surface had some adhesion to chest wall and visceral pleura of the lung. By blunt dissection and manipulation, both of the tumors were completely separated and removed. The tumors were approximately 40 × 25 × 15 cm and 28 × 25 × 10 cm in size, 3.78 and 2.85 kg in weight, respectively (Figs. 1C and 2C). Histologic examination revealed lipoma for the first patient, and well-differentiated liposarcoma for the second (Figs. 1D and 2D). Both of their symptoms were improved after surgery and the postoperative courses were good. There has been no evidence of recurrence for 40 months since the initial operation. This study was approved by the institutional review board of the Second Xiangya Hospital of Central South University. Informed consents were given by the patients.

## 3 Discussion

Lipomas or liposarcomas arising within the anterior mediastinum are quite uncommon tumors, constituting <2.5% of all primary mediastinal neoplasms.^[1,7,8]^ These tumors usually grow slowly, insidiously and remain asymptomatic until reach a huge size. Most of the symptoms such as dyspnea and dysphagia are due to tumor extend into both chest cavities or compress the adjacent structures. Schweitzer and Aguam reported that 85% of these kinds of patients had related symptoms, while other asymptomatic patients were diagnosed incidentally on a routine chest X-ray or CT examination.^[9]^

With similar attenuation on CT scan, both of these tumors showed a large mass with fat density and areas of soft-tissue density. Well-differentiated liposarcomas usually resemble lipomas. Fibrous septa may be thicker, more irregular, or more nodular than those seen in lipoma.^[10]^ Due to similar radiographic characteristics, preoperative differentiating a lipoma from a liposarcoma could be difficult in many cases, especially when it was a low-grade malignancy.^[11,12]^

Berry et al and Klimstra et al reported that the average size of liposarcoma was 15.7 cm, ranging from 6 to 40 cm, and the average weight was 1.5 kg. Such giant mass usually compress the intrathoracic organs, such as the heart, pericardium, great vessels, the lung, esophagus, and superior vena cava, causing life-threaten conditions.^[13,14]^ For the treatment, neither of lipomas nor liposarcomas is sensitive to chemotherapy or radiotherapy.^[15,16]^ Complete surgical excision using the standard median sternotomy or lateral thoracotomy is the most employed therapeutic choice.^[17]^ Subtotal resection is another acceptable choice but is of only short-term palliative benefit due to tumor recurrence despite postoperative adjuvant therapy. Aubert and colleagues reported metastasis and implantation of mediastinal liposarcoma after initial resection by the video-assisted thoracic surgery (VATS), indicating that VATS surgery may not be a good approach for the removal of malignant tumors of the mediastinum.^[18]^ Huang and Jiang reported complete removal of a giant mediastinal liposarcoma using a “
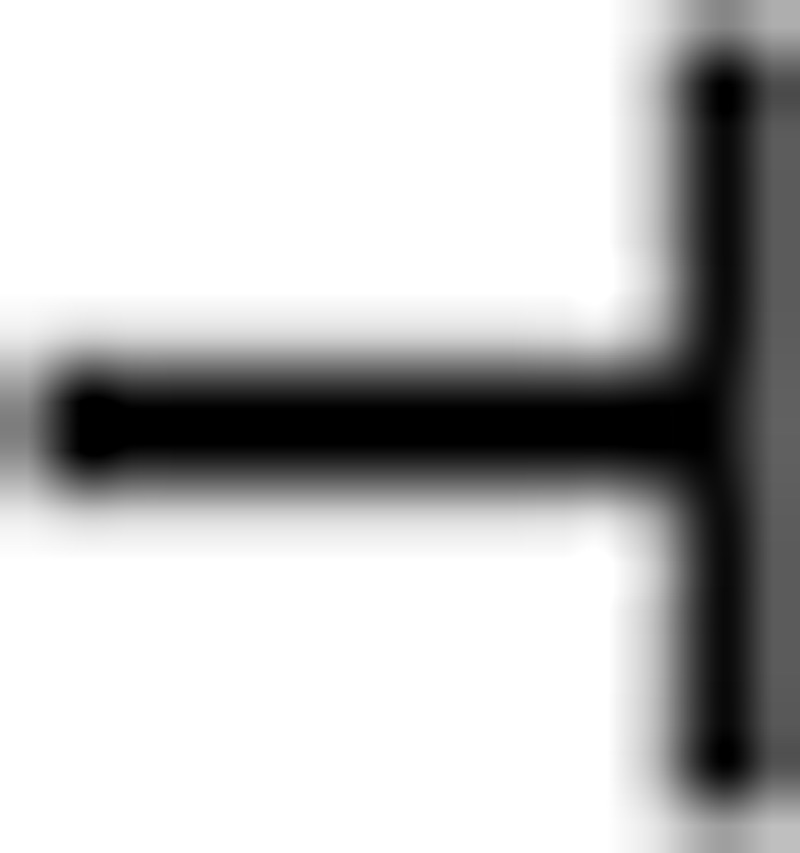
 shape” incision, indicting this kind of incision is a good backup for the extension of standard median sternotomy and provides a better exposure for both mediastinum and hemithorax.^[4]^

For our 2 patients, extended surgical removals were performed through a sternotomy to allow complete access to both chest cavities and the mediastinum. With sufficient exposure, both of the giant mediastinal tumors could be easily and completely removed. The postoperative courses were good. Our experiences on these 2 cases suggest that complete surgical resection through a standard median sternotomy is a safe and efficient approach for the treatment of giant mediastinal lipoma and liposarcoma.

## Author contributions

**Conceptualization:** Chen Chen, Yunchang Yuan.

**Data curation:** Wenliang Liu.

**Formal analysis:** Wenliang Liu.

**Investigation:** Chen Chen, Mingjiu Chen.

**Methodology:** Yunchang Yuan.

**Resources:** Yunchang Yuan.

**Writing – original draft:** Chen Chen.

**Writing – review & editing:** Fenglei Yu.
